# Dependence of Magnetic Properties of As-Prepared Nanocrystalline Ni_2_MnGa Glass-Coated Microwires on the Geometrical Aspect Ratio

**DOI:** 10.3390/s24113692

**Published:** 2024-06-06

**Authors:** Mohamed Salaheldeen, Valentina Zhukova, Ricardo Lopez Anton, Arcady Zhukov

**Affiliations:** 1 Department of Polymers and Advanced Materials, Faculty of Chemistry, University of the Basque Country, UPV/EHU, 20018 San Sebastian, Spain; valentina.zhukova@ehu.es; 2 Department of Applied Physics I, EIG, University of the Basque Country, UPV/EHU, 20018 San Sebastian, Spain; 3 Physics Department, Faculty of Science, Sohag University, Sohag 82524, Egypt; 4 EHU Quantum Center, University of the Basque Country, UPV/EHU, 20018 San Sebastian, Spain; 5 Department of Applied Physics, Regional Institute for Applied Scientific Research (IRICA), University of Castilla-La Mancha, 13071 Ciudad Real, Spain; ricardo.lopez@uclm.es; 6 IKERBASQUE, Basque Foundation for Science, 48011 Bilbao, Spain

**Keywords:** NiMnGa alloys, glass-coated microwires, magnetic field, Taylor–Ulitovsky technique, coercivity, microstructural properties

## Abstract

We have prepared NiMnGa glass-coated microwires with different geometrical aspect ratios, *ρ* = *d_metal_*/*D_total_* (*d_metal_*—diameter of metallic nucleus, and *D_total_*—total diameter). The structure and magnetic properties are investigated in a wide range of temperatures and magnetic fields. The XRD analysis illustrates stable microstructure in the range of *ρ* from 0.25 to 0.60. The estimations of average grain size and crystalline phase content evidence a remarkable variation as the *ρ*-ratio sweeps from 0.25 to 0.60. Thus, the microwires with the lowest aspect ratio, i.e., *ρ* = 0.25, show the smallest average grain size and the highest crystalline phase content. This change in the microstructural properties correlates with dramatic changes in the magnetic properties. Hence, the sample with the lowest *ρ*-ratio exhibits an extremely high value of the coercivity, *H_c_*, compared to the value for the sample with the largest *ρ*-ratio (2989 Oe and 10 Oe, respectively, i.e., almost 300 times higher). In addition, a similar trend is observed for the spontaneous exchange bias phenomena, with an exchange bias field, *H_ex_*, of 120 Oe for the sample with *ρ* = 0.25 compared to a *H_ex_* = 12.5 Oe for the sample with *ρ* = 0.60. However, the thermomagnetic curves (field-cooled—FC and field-heating—FH) show similar magnetic behavior for all the samples. Meanwhile, FC and FH curves measured at low magnetic fields show negative values for *ρ* = 0.25, whereas positive values are found for the other samples. The obtained results illustrate the substantial effect of the internal stresses on microstructure and magnetic properties, which leads to magnetic hardening of samples with low aspect ratio.

## 1. Introduction

Studies of alloys exhibiting thermoelastic martensitic phase transformations (TMPTs) represent a promising direction for various structural and functional applications due to their remarkable properties, including shape memory (SM), giant super elasticity (GS), magnetic field-induced strain (MFIS), and elastocaloric and magnetocaloric (EMC) effects [1,2,3,4,5,6,7,8,9,10,11,12,13,14,15,16,17,18,19,20]. These properties may be used for the development of actuators, sensors, energy harvesting devices, biomedical drug delivery pumps, and solar cells, among others [1,2,3,4,5,6,7,8,9,10,11,12,13,14,15,16,17,18,19,20,21,22].

Studies of Ni_2_MnGa alloys have recently gained significant attention due to their unique magnetic shape-memory effect (MSE). This effect provides both large field-induced strains and super elasticity exceeding traditional shape-memory alloys (SMAs), which are related to thermoelastic martensite transformation [13,14,15,16,17,18,19,20,21,22]. Conventional SMAs undergo reversible shape changes between austenite and martensite phases triggered by temperature or stress variations, enabling their use in actuators, sensors, and energy transducers requiring high-frequency responses and reversible strains. Miniaturization of SMA-based devices has increased the demand for small-scaled samples like particles, wires, ribbons, films, and microstructures [23,24,25,26]. However, NiMnGa alloys, typically intermetallic compounds, exhibit brittleness that hinders fabrication using traditional methods like cold drawing or forging [15]. While single crystals offer improved ductility, their production is time-consuming and prone to chemical segregation, compromising their performance [13,14,15,16,17]. The Taylor–Ulitovsky technique [27] offers a viable solution, enabling the production of glass-coated microwires with lengths reaching kilometers and diameters ranging from 0.1 to 100 µm [28,29]. This method provided high cooling rates, allowing the preparation of amorphous, nanocrystalline, microcrystalline, or granular structures in high-entropy states [28,29,30]. The unique combination of tunable magnetic properties together with improved mechanical and corrosion properties (linked to the existence of insulating and flexible glass-coating), low dimensionality, and reduced eddy current losses make such glass-coated microwires attractive for various magnetic sensor applications [28,29,30,31]. 

Previous studies successfully utilized rapid quenching methods to fabricate Heusler-type magnetic wires [30,31,32,33,34,35,36,37,38,39]. Notably thicker (226-µm diameter) glass-coated NiMnGa wires exhibited reversible super elasticity up to 4% tensile strain [40]. However, given the limitations of the preparation method, only rather short and fairly thick wires were obtained. Moreover, quite thick glass coatings are reported to affect mechanical properties and deteriorate the heat exchange rate [32,41,42]. While suitable for Heusler alloys with moderate Magnetocaloric Effect (MCE) and near-ambient Curie temperatures, the glass-coated technique often failed to exhibit martensitic transformation (MT) in Heusler microwires [30,31,32,33,34,35,36,37,38,39]. High internal stresses (100–1000 MPa), small grain size, and structural disorder associated with melt quenching are likely responsible [30,38]. Only recently, MT has been observed in properly annealed Ni-Mn-Ga glass-coated microwires [31,38]. Therefore, identifying appropriate fabrication conditions remains crucial to achieving MT and unlocking versatile properties in Heusler-type microwires.

Based on our previous research on the magnetic and structural properties of NiMnGa-based glass-coated microwires [30,31,35,37,38,39,41], this work aims to elucidate the influence of the aspect ratio on the magneto-structural behavior of as-prepared NiMnGa alloys without any further additional thermal process. We have observed how the main key magnetic parameters for sensing applications (coercivity, exchange bias, hysteretic behavior…) can be tuned just by controlling the microstructural properties by changing the geometric aspect ratio.

## 2. Materials and Methods

For Ni_2_MnGa alloy fabrication, we used precise arc melting, consisting of the following steps: (i) Ni, Mn, and Ga powders (99.99% purity) were weighed and placed in a graphite crucible. (ii) Melting occurred under vacuum/argon in an electric arc furnace with temperature control for complete melting and mixing. (iii) The crucible cooled after complete melting, solidifying into an ingot. This process was repeated five times to achieve optimal homogeneity and microstructure. The ingot was then used for the preparation of microwires coated with thin glass coatings below a few µm) preserving critical electrical/magnetic properties using the Taylor–Ulitovsky method.

The well-established Taylor–Ulitovsky process is described elsewhere [28,30,36,41]. The diameter of the metallic nucleus and the thickness of the glass coating can be controlled by adjusting several key parameters during the wire fabrication process, such as the speed at which the wire is drawn, the feed rate of the glass tube, or the ingot temperature [28,30]. In this study, we fabricated four distinct types of Ni_2_MnGa glass-coated microwires with varying geometrical characteristics, specifically the diameters of the metallic nucleus and the overall wire diameter. We achieved this variation by manipulating the aforementioned parameters, such as the drawing speed, the pick-up bobbin rotation speed, or the ingot temperature (see Table 1).

Energy Dispersive X-ray Spectroscopy (EDX) analysis confirmed the chemical composition of the metallic nucleus as Ni_59.2_Mn_12.2_Ga_28.6_ (atomic percent). Scanning Electron Microscopy (SEM) revealed a cylindrical cross-section with a notably homogeneous distribution of elements within the metallic nucleus. Notably, an interface layer was observed between the metallic nucleus and the surrounding glass coating. Furthermore, a BRUKER X-ray diffractometer (D8 Advance, Bruker AXS GmbH, Karlsruhe, Germany) was utilized to execute Cu Kα (λ = 1.54 Å) radiation for their structural investigation. All the magnetic measurements were performed using a PPMS (Physical Property Magnetic System, Quantum Design Inc., San Diego, CA, USA) vibrating-sample magnetometer. Thermo-magnetization measurements were performed in the temperature, *T*, range from 5 to 400 K with a magnetic field ranging from 10 kOe to 20 kOe (applied along the sample’s axis), whereas the hysteresis loops were obtained using a zero-field-cooled (ZFC) protocol at different temperatures in the previous range and with a maximum applied field of 30 kOe. All the magnetic results are presented in terms of normalized magnetization (M/M_max field_ or M/M_5K_) to account for the relative nature of the measurements, where M_5K (max. field)_ refers to the magnetic moment measured at 5 K or under maximum field, respectively. The Curie temperature, *Tc*, has been determined as the minimum of the first derivative of the magnetic moment.

## 3. Results

### 3.1. Morphological Properties of Ni_2_MnGa Samples

**Figure 1:**
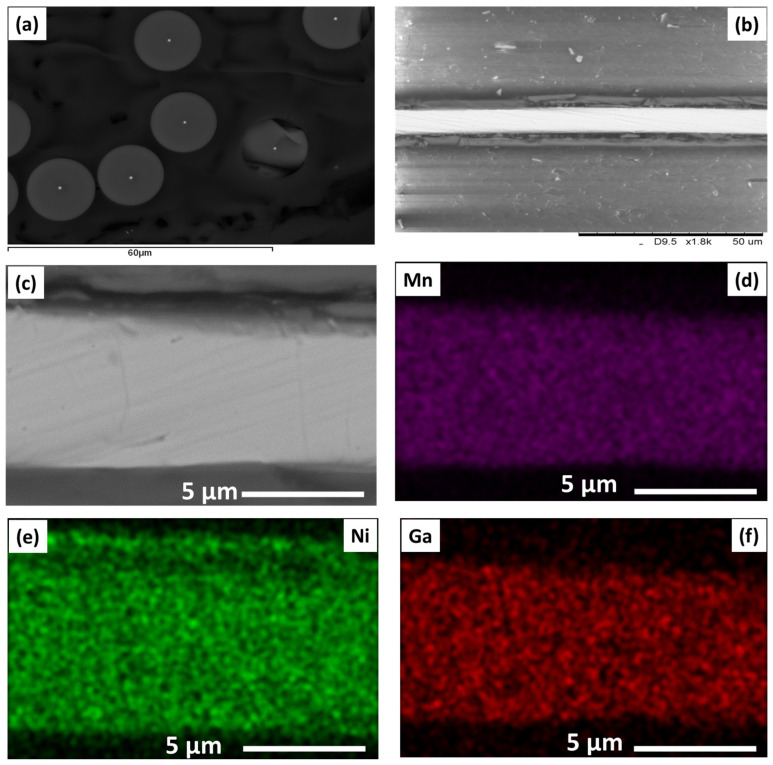
(**a**) Cross-sections of selected Ni_2_MnGa microwires with an aspect ratio of 0.25. (**b**,**c**) SEM image for single Ni_2_MnGa microwire at different magnifications. (**d**–**f**) show the chemical composition mapping obtained using EDX analysis in one of microwires.

To explore the influence of aspect ratio, *ρ*, on magnetic and microstructural properties of as-prepared Ni_2_MnGa glass-coated microwires produced using the Taylor–Ulitovsky process, four samples with different diameter *ρ*-ratios (*ρ* = *d_metal_*/*D_total_*, being *d_metal_*—diameter of the metallic nucleus, and *D_total_*—total microwire diameter) were chosen. The first sample (A) has an average metallic nucleus diameter (d _metal_) of ~6.77 μm and a total diameter (*D_total_*) of ~26.58 μm, i.e., aspect ratio *ρ* = 0.25. The second sample (B) has an average metal nucleus diameter (*d _metal_*) of ~31.60 μm and a total diameter (D _total_) of ~69.10 μm (*ρ* = 0.47). The other two samples have higher geometrical aspect ratios: *ρ* = 0.55 and 0.60, respectively (see Table 1). Energy-dispersive X-ray spectroscopy (EDX) combined with scanning electron microscopy (SEM) was used to determine the actual chemical composition of the Ni_2_MnGa- based glass-coated microwires with different aspect ratios (see Figure 1 and Table 1). Analysis of six different locations revealed average chemical compositions as follows: Ni_49_Mn_24_Ga_27_ (A), Ni_50.5_Mn_23_Ga_26.5_ (B), Ni_50_Mn_24.5_Ga_25.5_ (C), and Ni_50_Mn_24_Ga_26_ (D), respectively. These compositions are close to the intended stoichiometric ratio of 2:1:1 (Ni_50_Mn_25_Ga_25_) and confirm the consistency with the expected values (Table 1). The EDX elements mapping of all samples shows a homogeneous element distribution of Ni, Mn, and Ga. Therefore, we only show those compositional maps for the Ni_2_MnGa-MWs with the lowest aspect ratio as an example (see Figure 1c–f). 

### 3.2. XRD Analysis of Ni_2_MnGa-Based Glass-Coated Microwire Samples

X-ray diffraction (XRD) analysis was performed at room temperature on as-prepared Ni_2_MnGa glass-coated microwires with varying *ρ*-ratios. While the XRD patterns show quite similar diffractograms for all crystalline phases, subtle structural differences (peak intensity and position) are observed between microwires with different *ρ*-ratios. These variations are most pronounced in the analysis of the first peak, identified as (110) using the International Center for Diffraction Data (ICDD) database (https://www.icdd.com/, accessed on 23 February 2024). Gaussian fitting of the crystalline peak allows for the calculation of its area, proportional to the corresponding crystalline phase content.

The average crystalline grain size (D_g_) can be determined from the first peak’s position and width using the established Debye–Scherrer equation (Equation (1)):

D_g_ = Kλ/(B cos(θ)) (1)

where Dg represents the average crystallite size, K is the shape factor (assumed to be 0.9), λ is the X-ray wavelength (0.154 nm for Cu Kα1 radiation), B is the full width at half maximum (FWHM) of the corresponding diffraction peak in radians, and θ represents the Bragg angle.

**Figure 2:**
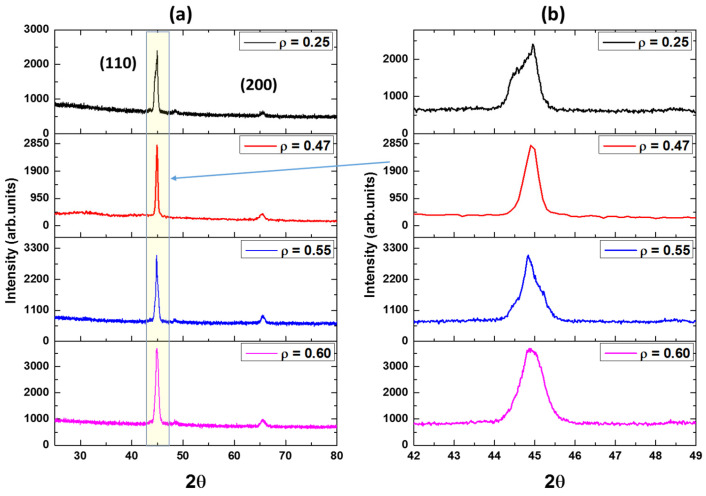
(**a**) X-ray diffraction (XRD) patterns obtained at room temperature for Ni_2_MnGa glass-coated microwires with varying aspect ratios. (**b**) Detail of X the Bragg Peak at *2θ* = 45° is shown as yellow area.

**Figure 3:**
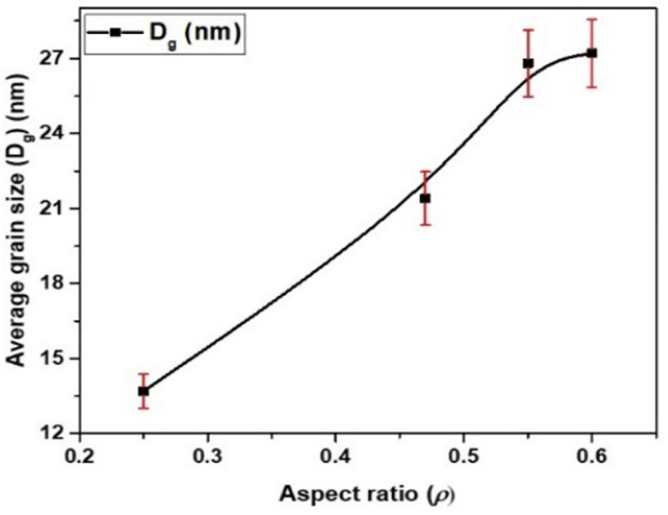
The average grain size, *D_g_*, of the two crystalline phases Ga_4_Ni_3_-BCC and Ni_2_MnGa-FCC of as-prepared Ni_2_MnGa-based glass-coated microwires with different aspect ratios. The red lines indicate the error bars.

As can be seen in Figure 2, all the samples show two main peaks with Miller indices (110) and (200) at *2θ* = 45° and 60°, respectively. These obtained by XRD data are perfectly matched with those reported elsewhere [42]. The main peak is at *2θ* = 45° with (110). These differences are strongly related to the variation in the geometrical parameters (*ρ*-ratio). Figure 3 illustrates the dependence of the average grain size of two phases, estimated using (1), of two peaks with miller parameters (110) and (200), respectively, on the geometrical aspect ratio, *ρ*. As we increase the geometrical aspect ratio, a notable increase in *D_g_* is observed, increasing from 13.6 nm (*ρ* = 0.25) up to 27.2 nm (*ρ* = 0.65).

Following the same procedures reported in our previous work (see [31,38]), we have analyzed the XRD peaks and determined the different phase structures. Two main phases, Ga_4_Ni_3_ (BCC) and Ni_2_MnGa (FCC), are identified from the XRD spectra, agreeing well with the results from references [31,38]. There is also a wide halo (from 15° to 22°; in Figure 2) due to the amorphous glass. Table 2 summarizes the variations of different structure phases and lattice parameters with the variation of the geometrical aspect ratios. As can be seen, there is a clear variation in the Ga_4_Ni_3_ and Ni_2_MnGa content due to changes in the geometrical aspect ratio. The sample with the smallest *ρ*-ratio shows the highest Ni_2_MnGa-FCC content (about 78%) and the lowest percentage of Ga_4_Ni_3_-BCC (22%). Meanwhile, the samples with high *ρ*-ratios (0.55 and 0.60) show the highest Ga_4_Ni_3_-BCC (83% and 81%) and lowest Ni_2_MnGa-FCC (17% and 19%), respectively. The changes in these microstructure parameters affect the magnetic behavior of the samples at different temperatures and different external magnetic fields, as will be discussed in the next section. 

### 3.3. Magnetic Properties of Ni_2_MnGa Samples

**Figure 4:**
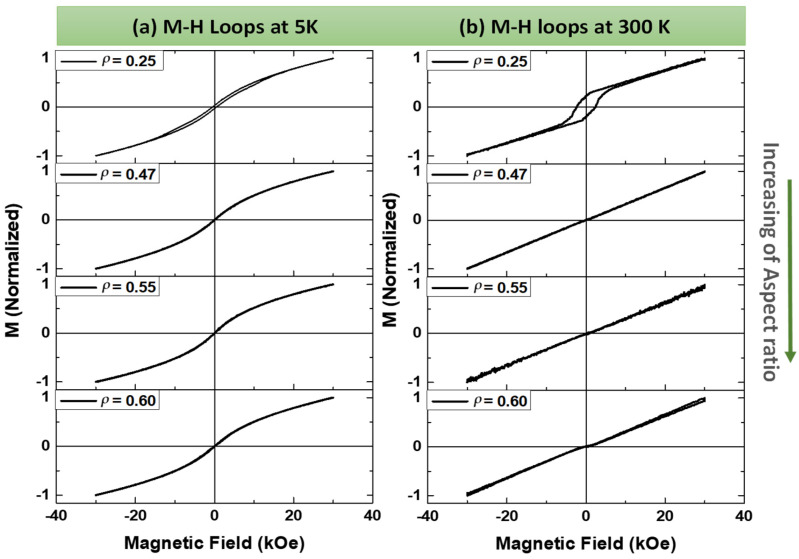
(**a**) Hysteresis loops, measured in magnetic field applied parallel to the axis of microwires for as-prepared Ni_2_MnGa glass-coated microwires with different *ρ*-ratios (**a**) measured at 5 K and (**b**) measured at 300 K.

Figure 4 presents the magnetization (M)-versus-magnetic field (H) loops for samples with different *ρ*-ratios, measured at both *T* = 5 K (Figure 4a) and *T* = 300 K (Figure 4b). Interestingly, the sample with the smallest *ρ*-ratio exhibits unexpected magnetically hard behavior, characterized by high coercivity and remanence. In contrast, the remaining microwires display soft ferromagnetic behavior, as evidenced by the *M-H* loops measured at *T* = 5 K and the pronounced paramagnetic behavior observed in the *M-H* loops measured at *T* = 300 K. These latter loops appear nearly linear with negligible remanence and coercivity. The observed *M-H* behavior suggests a link between the geometrical aspect ratio and the magnetic properties of the metallic nucleus in these glass-coated microwires. This link is further supported by the apparent changes in Curie temperature (*Tc*). Our results indicate that the *Tc* of the sample with *ρ* = 0.25 lies above room temperature (RT), whereas the *Tc* of the other samples falls below RT. It is noteworthy that the Curie temperature of the NiMnGa alloy is rather sensitive to factors such as chemical composition, preparation method, structure, physical form, and heat treatment [30,38]. Generally, the Curie temperature for this alloy can range between 160 K and 360 K.

In our experiment, even though all NiMnGa-based glass-coated microwires were prepared using the same process and exhibit very similar chemical compositions (as shown in Table 1), a clear shift in *Tc* is observed between the samples. This suggests that the microstructure of the sample plays a significant role in determining the *Tc* value and, as will be discussed later, the overall magnetic behavior.

**Figure 5:**
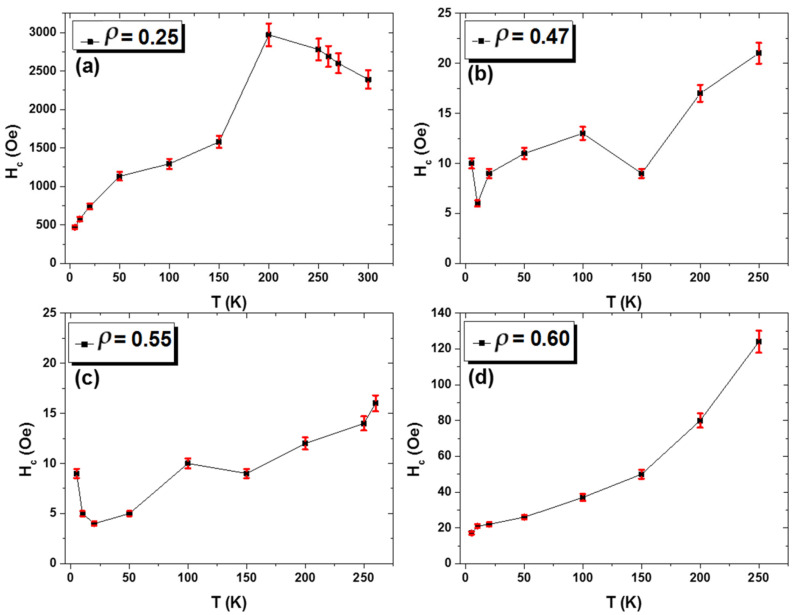
(**a**–**d**) The variation of coercivity with temperature for Ni_2_MnGa-based glass-coated microwires with different aspect ratios varied from 0.25 to 0.60. Red lines indicate the error bars.

The *H_c_*(*T*) dependencies for all the samples are summarized in Figure 5. The sample with *ρ* = 0.25 shows the highest *H_c_*-values for all measured temperatures, where the highest *H_c_*-value (*H_c_* = 2989 Oe) is observed at *T* = 200 K and the lowest *H_c_* ≈ 480 Oe is observed at *T* = 5 K (see Figure 5a). The *H_c_*-value for the sample with *ρ* = 0.25 is 300 times higher than the *H_c_* for *ρ* = 0.47 and *ρ* = 0.55 and 75 times higher than for the sample with *ρ* = 0.60 measured at the same temperature (*T* = 200 K). In addition, the *H_c_*(*T*) dependence is substantially affected by *ρ*-ratio. Hence, a coercivity maximum is observed at *T_f_* = 200 K for the sample with *ρ* = 0.25 while, in contrast, a decrease in *H_c_* is generally observed as *T* decreases for the other three microwires. In particular, a monotonic decrease in *H_c_*-value while decreasing the *T* from 265 K to 5 K is obtained for the sample with *ρ* = 0.60 (see Figure 5d), whereas for the samples with *ρ* = 0.47 and *ρ* = 0.55, a local maximum at ≈100 K and an increase in *H_c_*-value at *T* ≤ 20 K are observed, respectively.

**Figure 6:**
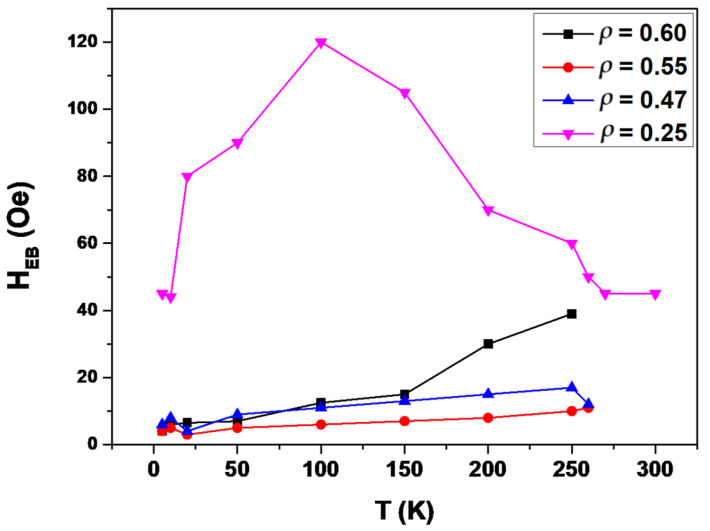
Temperature dependence of the spontaneous exchange bias for Ni_2_MnGa glass-coated microwires with different aspect ratios (lines for eye guide).

Figure 6 shows the dependence of the spontaneous exchange bias field, *H_EB_*, vs. temperature for all the samples. The observation of *H_EB_* is quite common in NiMnGa alloys and is attributed to the coexistence of antiferromagnetic and ferromagnetic phases in different range temperatures [28,31]. In our case, it is noteworthy that we observe spontaneous exchange bias (i.e., we observe these phenomena even though the measurements were performed following a ZFC protocol) and with values quite similar to those found in other cases for microwires of NiMnGa alloys but following an FC protocol [31]. As seen in Figure 6, the sample with *ρ* = 0.25 shows the highest spontaneous exchange bias value, H_EB_, compared to the rest of the microwires. In addition, the *H_EB_* depends on the temperatures and shows a clear maximum of around 100 K. The *H_EB_*-value for (*ρ* = 0.25) is ≥12 times higher than the *H_EB_*-value observed in the other samples. The temperature dependencies of *H_EB_* for samples with *ρ* =0.47 and 0.55 show a similar tendency, although with a maximum fairly less marked and at quite low temperatures (at about 10 K). Meanwhile, two different slopes in the *H_EB_*(*T*) dependence are observed for the sample with *ρ* = 0.60, where *H_EB_* decreases rapidly as the temperature decreases from 265 to 150 K compared to a less pronounced *H_EB_* decrease for temperatures ranging from 150 to 5 K. Given these remarkable results of the spontaneous exchange bias, we intend to perform additional studies focusing on the conventional exchange bias for these samples in the near future. 

These changes in the *H_c_* and *H_EB_* behavior with temperature confirm, again, the substantial effect of the geometrical aspect ratios on the magnetization reversal process through the changes in the internal stress value induced by the glass-coating.

**Figure 7:**
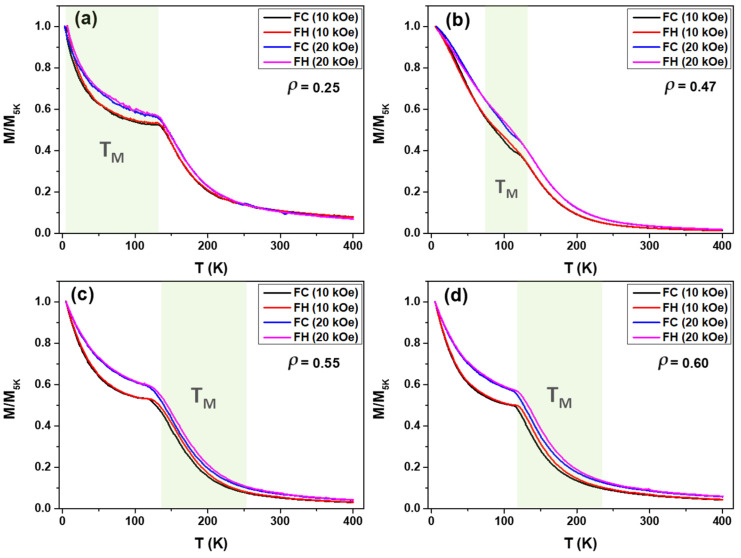
(**a**–**d**) Field cooling (FC) of Ni_2_MnGa glass-coated microwires at temperature range 400 K to 5 K with different applied magnetic field H = 10 kOe to 20 kOe. The green area points out the region where the T_M_ is expected to be observed.

To gain a deeper understanding of the magnetic behavior of the samples, thermo-magnetization measurements were performed under two different protocols: field cooling (FC) and field heating (FH). In FC, the samples were cooled from 400 K to the lowest temperature (5 K) under different applied fields (H) of 10 kOe and 20 kOe. The FH protocol involved the reverse process at the same H values. The FC and FH curves for all the samples are presented in Figure 7. For samples with *ρ* = 0.25, 0.55, and 0.60, the *M*/*M_5K_* (*T*) dependencies exhibit a non-monotonic decrease with temperature (see Figure 7a,c,d) with a knee observed at *T* ≈ 136 K for both applied fields. Similar behavior was previously observed in as-prepared NiMnGa-based glass-coated microwires and thin films [31,33,35,39,43,44,45,46,47]. Such *M*/*M_5K_* (*T*) dependencies and lack of saturation in *M-H* loops (see Figure 4) have been discussed in terms of a nonuniform magnetic character, the atomic disorder, and the magnetic clustering of the as-prepared microwires [32,39]. Meanwhile, for the sample with *ρ* = 0.47, the temperature dependence of the magnetic moment, *M*/*M_5K_* (*T*) (see Figure 7b), shows a small and narrow hysteretic anomaly between field cooling (FC) and field heating (FH) curves. A subtle change (hysteretic anomaly) is observed in the *M*/*M_5K_* (*T*) dependencies of studied microwires at a specific temperature range (85–120 K) regardless of the applied magnetic field (H). This anomaly suggests the presence of a martensitic transformation in the microwires. However, the difference in magnetizations between the martensitic and austenitic phases is small, which does not lead to a noticeable shift in the transformation temperature under changes in magnetic fields. The temperature for martensitic transition, *T_M_*, is below 120 K for samples with small *ρ*-ratios, i.e., 0.25 and 0.47, while for samples with higher aspect ratios, i.e., 0.55 and 0.60, *T_M_* is above 120 K (see green highlights in Figure 7). The *M*/*M_5K_* (*T*) dependencies (Figure 7) and *M*-*H* loops (Figure 4) reflect a nonuniform magnetic character of the as-prepared Ni_2_MnGa-based glass-coated microwires with different *ρ*-ratios produced by the atomic disorder and magnetic clustering. Despite the smeared shape of *M*/*M_5K_* (*T*) dependencies, for the sample with *ρ* = 0.25, it can be estimated that *Tc* is above room temperature (*Tc* ≈ 363 K). Meanwhile, for the rest of the NiMnGa microwires, *T_C_* varies between 250 and 265 K. However, above 130 K all the samples present weak ferromagnetic behavior. 

## 4. Discussion

The Taylor–Ulitovsky method, used to fabricate glass-coated microwires, is challenging due to the rather different thermal expansion coefficients of the involved materials (metallic alloy and glass-coating). The employed technique involves rapid solidification of a molten metal nucleus surrounded by a glass coating. The significant difference in thermal expansion coefficients between the metallic alloy and glass leads to the onset of internal stresses distributed in a complex manner within the metallic nucleus. Theoretical calculations and indirect experiments suggest these stresses can reach peak values between 100 and 1000 MPa [31,36], being considerably higher than the stresses exerted by external magnetic fields (1–7 MPa). These substantial internal stresses, caused by the thermal expansion mismatch, hinder crucial properties like martensitic transformation characterized by hysteresis behavior and magnetic response to temperature and magnetic fields. There is a strong correlation between glass coating thickness and internal stress magnitude, with thicker coatings leading to higher internal stress levels, as reported for microwires with lower *ρ*-ratios [36,48,49,50]. Furthermore, the impact of internal stress on the crystallization process can be understood through the lens of non-equilibrium thermodynamics: stress influences atomic diffusion, thereby affecting crystal nucleation and growth [51]. Recent studies on Heusler-alloy glass-coated microwires provide experimental evidence for this effect, as the recrystallization process differs significantly between glass-coated and uncoated samples [52,53,54]. Additionally, the presence of an insulating glass coating can affect the heat exchange rate and, hence, the quenching rate during the preparation of microwires by the Taylor–Ulitovsky method [28,29,30]. The quenching rate can affect the average grain size. However, we cannot observe a direct correlation between the *D_g_*-values and glass coating thickness (see Table 2). Therefore, we must assume that the main factor influencing the magnetic and microstructural properties of studied microwires is the internal stresses’ magnitude and distribution. In the current study, the modification in the internal stress by changing the *ρ*—ratios induces a variety of microstructure properties, where variations in the values of the average grain size and the crystalline phase content are observed. Furthermore, changing the geometrical substantially affects the magnetic behavior. The sample with *ρ*-ratio = 0.25 shows a rather hard magnetic behavior with a coercivity value near 3 kOe. In this sample, the stronger ferromagnetic behavior around the room temperature must be related to the higher Ni_2_MnGa crystal phase content (78%). Meanwhile, for the rest of NiMnGa microwire samples with *ρ*-ratios above 0.25, the paramagnetic behavior is observed above T = 265 K due to the lower content of ferromagnetic phase, Ni_2_MnGa and higher paramagnetic Ga_4_Ni_3_ crystalline phase content (see Table 2). Thus, in NiMnGa glass-coated microwires with different geometrical parameters, the variation of the content of different phases affects temperature and magnetic field dependencies of magnetic properties. Therefore, the tunability of microstructure and magnetic properties of glass-coated Ni_2_MnGa microwires to change in geometrical aspect ratio, demonstrated by the microstructural–magnetic investigation, make them an appropriate candidate for use as sensing materials.

## 5. Conclusions

In summary, we report the possibilities to tailor the microstructure and magnetic properties of the as-prepared NiMnGa glass-coated microwires by using the Taylor–Ulitovsky method by only changing its geometrical aspect ratios without needing additional annealing treatment. The microstructure analysis proves the strong dependence of the average grain size and crystalline phase content on the geometrical aspect ratio. In addition, the existence of two main phases, a ferromagnetic (Ni_2_MnGa) one and a paramagnetic (Ga_4_Ni_3_) one, with changing contents depending on the aspect ratio, allows the control of the magnetic behavior of the NiMnGa glass-coated microwires. For the microwire with high ferromagnetic phase content, high Curie temperature, coercivity (≈3 kOe), remanence, and spontaneous exchange bias are observed. Meanwhile, the microwire samples with low ferromagnetic phase content exhibit soft magnetic behavior with coercivity values about 10 Oe and Curie point below RT. Additionally, the microstructure (likely related to the presence of internal stresses) also affects the thermomagnetic behavior of samples. 

## Figures and Tables

**Table 1:** Chemical compositions and geometrical parameters of Ni_2_MnGa glass-coated microwire forms with different aspect ratios.

Sample	Chemical Composition	*D_total_* (µm)	*d_metal_* (µm)	Aspect Ratio (*ρ*)
A	Ni_49_Mn_24_Ga_27_	26.58	6.77	0.25
B	Ni_50.5_Mn_23_Ga_26.5_	69.10	31.6	0.47
C	Ni_50_Mn_24.5_Ga_25.5_	24.33	13.4	0.55
D	Ni_50_Mn_24_Ga_26_	21.07	12.20	0.60

**Table 2:** The calculations of the crystalline phase content, lattice parameters, and related grain size for Ni_2_MnGa glass-coated microwires with different aspect ratios.

Aspect Ratio (*ρ*)	Ga_4_Ni_3_-BCC			Ni_2_MnGa-FCC		
	*D_g_* (nm)	%	Lattice Parameter (a)	*D_g_* (nm)	%	Lattice Parameter (a)
0.25	9.7	22	0.235 nm	16.1	78	0.309 nm
0.47	26.9	68	0.286 nm	27	32	0.244 nm
0.55	25.5	83	0.204 nm	24	17	0.242 nm
0.60	27.8	81	0.221 nm	26.9	19	0.241 nm

## Data Availability

The data presented in this study are available upon request from the corresponding author.
